# Trans-generational responses to low pH depend on parental gender in a calcifying tubeworm

**DOI:** 10.1038/srep10847

**Published:** 2015-06-03

**Authors:** Ackley Lane, Camilla Campanati, Sam Dupont, Vengatesen Thiyagarajan

**Affiliations:** 1 The Swire Institute of Marine Science and School of Biological Sciences, The University of Hong Kong, Hong Kong SAR; 2 Department of Biological and Environmental Sciences, The Sven Lovén Centre for Marine Sciences - Kristineberg, University of Gothenburg, Fiskebäckskil, Sweden

## Abstract

The uptake of anthropogenic CO_2_ emissions by oceans has started decreasing pH and carbonate ion concentrations of seawater, a process called ocean acidification (OA). Occurring over centuries and many generations, evolutionary adaptation and epigenetic transfer will change species responses to OA over time. Trans-generational responses, via genetic selection or trans-generational phenotypic plasticity, differ depending on species and exposure time as well as differences between individuals such as gender. Males and females differ in reproductive investment and egg producing females may have less energy available for OA stress responses. By crossing eggs and sperm from the calcareous tubeworm *Hydroides elegans* (Haswell, 1883) raised in ambient (8.1) and low (7.8) pH environments, we observed that paternal and maternal low pH experience had opposite and additive effects on offspring. For example, when compared to offspring with both parents from ambient pH, growth rates of offspring of fathers or mothers raised in low pH were higher or lower respectively, but there was no difference when both parents were from low pH. Gender differences may result in different selection pressures for each gender. This may result in overestimates of species tolerance and missed opportunities of potentially insightful comparisons between individuals of the same species.

As seawater takes up anthropogenic CO_2_ emissions the pH and carbonate ion concentrations are reduced, a process called ocean acidification (OA)^1^. For most marine invertebrates, OA will be experienced through many generations allowing for trans-generational phenotypic plasticity (TPP) and genetic selection to act^2^,^3^,^4^. Trans-generational responses (i.e. TPP and/or genetic selection) appear to depend on species, environmental evolutionary history, exposure time and differences between individuals but much remains to be explored^5^,^6^. Gender, for example, an easily identifiable characteristic that divides species into two groups, can determine how an individual responds to or is affected by change in pH^7^. Differential investment in reproduction may determine intrinsic tolerance levels^8^, possibly influencing an individual’s fitness via the performance of their offspring. As of yet there are very few studies that explicitly examine the differences in stress tolerance between genders. Here, by crossing eggs and sperm from calcareous serpulid polychaete tubeworms raised in two pH environments, we observed that parental gender interacted with parental pH environment to affect offspring performance. The influence of males and females raised in low pH environments on offspring performance (i.e. metamorphosis and post-settlement growth rate) were opposite and additive, low pH males increased performance while low pH females decreased offspring performance. Intrinsic differences between males and females may result in different selection pressures or TPP strategies and while they may be hidden in nature, may shape species response to environmental change over time.

Working toward the ultimate goal of anticipating the effects of OA, most experimental designs use acute exposure of extant marine invertebrates to “future ocean” conditions^2^. Despite the potentially misleading results of acute experiments, very few studies have considered adaptive plasticity or evolutionary adaptation^2^,^9^. A few studies have found standing genetic variation in tolerance to low pH^10^,^11^,^12^,^13^. It has also been shown that low pH can be an evolutionary force by selecting certain genotypes^14^ and some have demonstrated that existing populations can exhibit pre-adaptation to low pH conditions^15^,^16^,^17^,^18^. The influence of low pH during the parental life can also influence offspring performance and studies have found positive (e.g. oysters^19^ and fish^20^,^21^), neutral (e.g. barnacles^22^) and negative (e.g. urchins^5^,^23^) trans-generational effects. The possibility of adaptation to environmental change over time is clearly demonstrated as standing genetic variation exists, rapid adaptive evolution is possible, differences in pH tolerance between populations exist and parental experience can alter an offspring’s phenotype^5^,^10^,^11^,^12^,^14^,^15^,^16^,^17^,^19^,^20^,^21^,^22^,^23^.

Rarely considered are the intrinsic differences in how males and females experience different pH environments^24^. Ellis, *et al.*^7^ demonstrated the importance of gender in pH response as the metabolome of males and females of the mussel *Mytilus edulis* differed significantly under stress. Reproductive costs are at the root of differences between males and females in anisogamous systems, with females commonly allocating more energy into reproduction than males^25^. For example, male and female polychaete worms of the genus *Ophryotrocha* (Dorvilleidae) can have different energy budgets with males investing less in reproduction and growing faster while females invest more in reproduction and grow less^26^. Responding to and coping with stress is energetically expensive and may demand a reworking of the organisms’ energy budget^27^, which may already be stretched, particularly for females. Reproduction strategies in sessile, broadcast spawning invertebrates tend to maximize maternal fitness and not the fitness of any individual offspring. It is therefore likely that females will prioritize maintenance of their own body function over reproductive investment^28^. Thus, it can be hypothesized that performance of females exposed to environmental change (e.g. OA), and ultimately their ability to reproduce and the quality of their offspring, will be more strongly affected than males. To date no study has examined the role that parental gender has in determining the subsequent generation’s responses when exposed to OA conditions.

Here, trans-generational responses (i.e. TPP and/or genetic selection) to OA were examined by exposing both parents and offspring of the biofouling tube worm *Hydroides elegans* (Haswell, 1883) to two pH environments within their natural range, a high pH (8.1) environment and a low pH (7.8) environment. This species is well suited to laboratory experiments due to the ease of culture and relatively short generation times^29^ and previous experiments have shown that tube growth and calcification can be affected by low pH conditions^30^,^31^,^32^. We tested the effects of and interactions between the pH environment experienced by the parents (from hatching to sexual maturation, F_0_ generation), parental gender, and the pH experienced by the offspring (F_1_ generation, experimental design summarized in Fig. 1) on F_1_ generation performances (i.e. metamorphosis success, juvenile growth and juvenile survival). We hypothesized that (i) offspring exposed to the same pH condition as that experienced by their parents will perform relatively better than those exposed to a different pH environment and (ii), because females may be more strongly affected by low pH conditions, possibly due to a tighter energy budget, they may experience stronger selection pressure or be less able to pass on positive trans-generational phenotypic changes.

## Results

### Larval metamorphosis

Metamorphosis success (%) was measured in both F_0_ and F_1_ generations (Fig. 2a). More than 50% of all the larvae successfully metamorphosed in the F_1_ generation. In the F_0_ generation this parameter was significantly affected by block (F = 19.29, p < 0.0001) but unaffected by pH (p > 0.05) or the pH by block interaction (p > 0.05). In the F_1_ generation metamorphosis success was significantly impacted (3-ways GLM model, F_7,35_ = 2.31, p = 0.048) by the pH experienced of the F_0_ females (F = 4.72, p = 0.037), F_1_ generation pH (F = 4.33, p = 0.045) and their interactions (F = 5.37, p = 0.026). The pH experienced of the F_0_ males and all other multiple factor interactions were not significant (p > 0.05). When the F_0_ females were exposed to pH 7.8, the metamorphosis success of offspring exposed to pH 8.1 was decreased by 30% as compared to other treatments.

### Post-metamorphic (juvenile) survival

Juvenile survival (in %) was assessed between day 1 and day 7 post-metamorphosis assay (Fig. 2b). In F_0_, pH had a significant effect with juvenile raised at pH 7.8 experiencing a 20% decrease in survival (2-ways GLM model, F_5,119_ = 19.47, p < 0.0001; pH, F = 4.55, p = 0.035) A significant block effect, due to multiple collections of wild adults for spawning, was also detected (F = 44.37, p < 0.0001) but with no interaction with pH (F = 40.58, p = 0.56). Similar survival around 60% was observed in F_1_ but none of the tested parameters (pH treatment, and pH experienced by the parents) had a significant effect (3-ways GLM model, F_6,29_ = 1.37, p = 0.26).

### Post-metamorphic (juvenile) growth

Seawater pH had no significant effect (2-ways GLM model, F_5,685_ = 303.81, p < 0.0001; pH, F = 3.07, p = 0.095; block, F = 908.18, p < 0.0001; pH × block, F = 0.17, p = 0.98) on F_0_ juvenile growth rate (Fig. 2c). For F_1_, the pH experienced by males (3-ways GLM, model, F_7,17_ = 5.30, p = 0.0024; males, F = 6.21, p = 0.023) and female parents (F = 7.39, p = 0.014) but also the interaction between males and pH (F = 16.67, p = 0.0008) had an impact on the juvenile growth rates. The F_1_ pH and other interactions were not significant (p > 0.05). When F_0_ males were exposed to pH 7.8 the F_1_ juvenile growth rate is increased 2 times when raised at pH 8.1 (e.g. compare F8.1_M8.1 to F8.1_M7.8 in pH 8.1 environment). On the other hand, the F_1_ growth rate is decreased by 25% when the F_0_ female parents were exposed to pH 7.8 (e.g. F8.1_M8.1 compared to F7.8_M8.1).

## Discussion

Both maternal and paternal low pH experience affected F_1_ growth rate, and maternal low pH experience affected metamorphosis success, however all differences between groups of the F_1_ generation (i.e. due to parental environment and gender) were only apparent when raised at high pH. The differences observed between the F_0_ and F_1_ generations are notable, and the reason behind the lower performance of the F_0_ generation is unknown but may be because the wild adults’ environment was unstable with an unpredictable food supply while the laboratory raised parental generation (F_0_) had a constant food supply. To summarize, when the eggs came from individuals raised in low pH environments, F_1_ metamorphosis and growth rate in the high pH environment were reduced by 30% and 25% respectively. On the other hand, when sperm came from individuals raised in low pH conditions, F_1_ growth rate in high pH conditions was more than doubled. Survival data, however, suffered from high variability and no significant difference between groups was observed in the F_1_ generation. So, parental gender and pH experience influenced the F_1_ generation confirming that there was a trans-generational response, although the majority of the effects were only apparent when F_1_ individuals were raised in high pH conditions. It is of particular interest that F_0_ females may be more strongly affected by low pH than F_0_ males, as F_0_ low pH females, unlike F_0_ low pH males, produced offspring whose metamorphosis success and growth rates decreased under high pH conditions. The fitness of each gender may be differently affected, and a species mean tolerance (without the consideration of gender) may actually overestimate the actual species tolerance as both genders are equally important in successful reproduction.

This experimental design highlights that parental influence, positive, negative, or neutral can result from a combination of paternal and maternal influence. If examining only F_1_ groups whose parents experienced the same pH environments (i.e. F8.1_M8.1 and F7.8_M7.8) one would conclude that there were no significant differences when parents are raised in either low or high pH. However disentangling parental gender by examining F_1_ groups where parents experience different pH environments (i.e. F7.8_M8.1 and F8.1_M7.8) shows that the ostensible neutrality of the F_0_ exposure (i.e. no differences between groups F7.8_M7.8 and F8.1_8.1) is a product of opposite and additive influences of each parental gender. While in nature genders are unlikely to experience different pH environments, the intrinsic experiences of each gender and their effect on the next generation may provide insight into intra-specific variation and highlight particular and gender-specific mechanisms that are important when responding to pH change^24^. Differences between genders stem from differences in reproductive investment, sperm is cheap while eggs are expensive^25^,^26^. Females may, therefore, have less spare energy to invest in somatic growth or plastic responses that would allow survival and reproduction in changing environments^33^. Further support for this hypothesis is given by Holcomb, *et al.*^8^ where high CO_2_ impacted calcification only in females (at increased temperature). The long term and potentially evolutionary, consequences due to differences between genders , will be hidden as a population’s phenotype changes over time unless specifically investigated. Nonetheless, the role gender differences play in evolution may be substantial as a female’s tighter energy budget may put them closer to the edge of their tolerance, resulting in stronger selection pressure on female characteristics as environments change.

The mechanisms responsible for the differences observed in the F_1_ generation cannot be established from the data presented. Evolutionary selection or trans-generational phenotypic plasticity (TPP) may have acted individually or simultaneously. Evolutionary selection and TPP work via entirely different mechanisms, either selecting more fit phenotypes that are genetically determined (inheritable) or by the ability to pass epigenetic information, energetic advantage via differentially egg provisioning or hormonal signals to offspring^34^. Each process could explain the environment-phenotype mismatch when F1 low pH females produced offspring with lower performance in high pH conditions. Likewise, F1 low pH males produced offspring with increased growth rates under high pH conditions, which could also be an environment-phenotype mismatch depending on whether faster growth is advantageous. Finally, differential maternal provisioning as a general increase or decrease in egg resources, which would exist in both F_1_ environments, is unlikely because the differences among F_1_ groups were only apparent in high pH treatments. However, advantageous natural selection or TPPs may have counteracted poor egg quality exclusively in the low pH environment.

Despite the ambiguity of the mechanisms behind the parental effects on the responses in the F_1_ generation, gender may determine stress tolerance in an individual, possibly because of energy differences. The intrinsic differences in how each gender experiences environmental change became apparent when the effects of males and females from each environment were considered separately, and not as a single pre-exposed unit. Rarely considered are the differences between sexes and pH tolerance, but further research into the genetic, transcriptomic, proteomic or metabolomics levels may provide insight into the gender specific mechanisms that are vital to survival and reproduction as environments change^7^. The opposite and additive influences of each parental gender on offspring observed here may play an important role in evolution as environments change, but also may provide interesting comparisons between highly related, but fundamentally different individuals. Low pH may be a relatively novel stress for which this species has no adapted response, for example parental effects in low salinity environments proved to be positive for both genders in the tube worm *Hydroides diramphus*^4^. The different influence of each gender due to low pH may identify specific characteristics or adaptive strategies beneficial in low pH seawater, giving a more complete picture when predicting species response as OA shifts oceanic carbonate chemistry. A more flexible energy budget may link directly to performance under low pH conditions and translate into stronger natural selection on females than males. Finally, knowing the how each gender is affected by changes in pH will give a more detailed understanding of species tolerances to low pH.

## Methods

The tube dwelling serpulid polychaete *Hydroides elegans* is a common biofouler that has been identified as a model species due to its economic and ecological importance, as well as its relatively short generation time (i.e. 2 weeks to maturity) and ease of culture^29^. *H. elegans* is capable of inhabiting highly variable habitats, like the Eastern coastal waters of Hong Kong, that vary in pH (8.3 to 7.6), temperature (16 to 29 °C) and salinity (30–33)^35^. For this multi-generational experiment wild adults were collected from the Eastern waters of Hong Kong, China field on 3 occasions and held in laboratory for no longer than 1 week before spawning. Wild adult worms were induced to spawn by gently breaking them from their tubes, those bearing mature gametes spawn immediately (3 males and 3 females on each spawning occasion, referred to as blocks). Within one hour of gamete collection eggs and sperm were combined at sperm concentrations of approximately 1 × 10^5^ sperm ml^−1^, shown to result in >90% fertilization and very little polyspermy^36^ The fertilized embryos (F_0_ generation) were then divided among replicates of two pH levels in culture tanks of 120 ml with 200–240 larvae in each (3 blocks × 2 pH levels × 54 replicates = 324 total replicate tanks, actual replication varied as some replicates did not meet the minimum requirements for inclusion). Filtered seawater in culture tanks was changed every other day and aliquots of concentrated algae cultures (*Isochrysis galbana*) were added to achieve approximate cell densities of 50,000 cells ml^−1^. All F_0_ generation animals used here were maintained for at least 3 months, from fertilization into maturity, in their respective treatments. The tubes of F_0_ individuals were all >2 cm when gamete spawning for the F_1_ generation was conducted.

**Figure 1:**
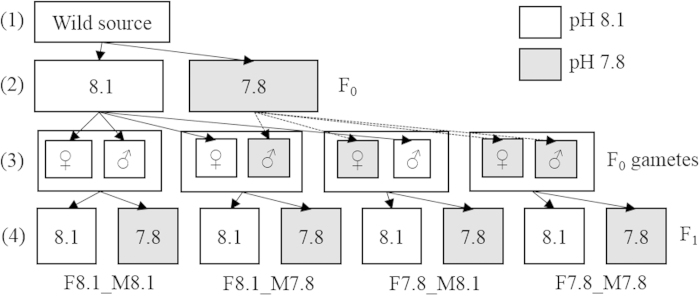
Experimental design. (**1**) Wild adults collected and spawned, and gametes were fertilized. (**2**) Larvae from wild adults were divided among two pH environments and raised to maturity (i.e. F_0_). (**3**) Gametes from F_0_ females (eggs) and males (sperm) of both pH environments were collected and fertilized in all possible combinations, creating 4 groups. (**4**) Each of the groups (F_1_ generation) were then divided among high and low pH environments and larval metamorphosis, juvenile growth and juvenile survival were measured (4 F_1_ groups × 2 pH environments × 6 replicates = 48 total F_1_ culture tanks).

**Figure 2:**
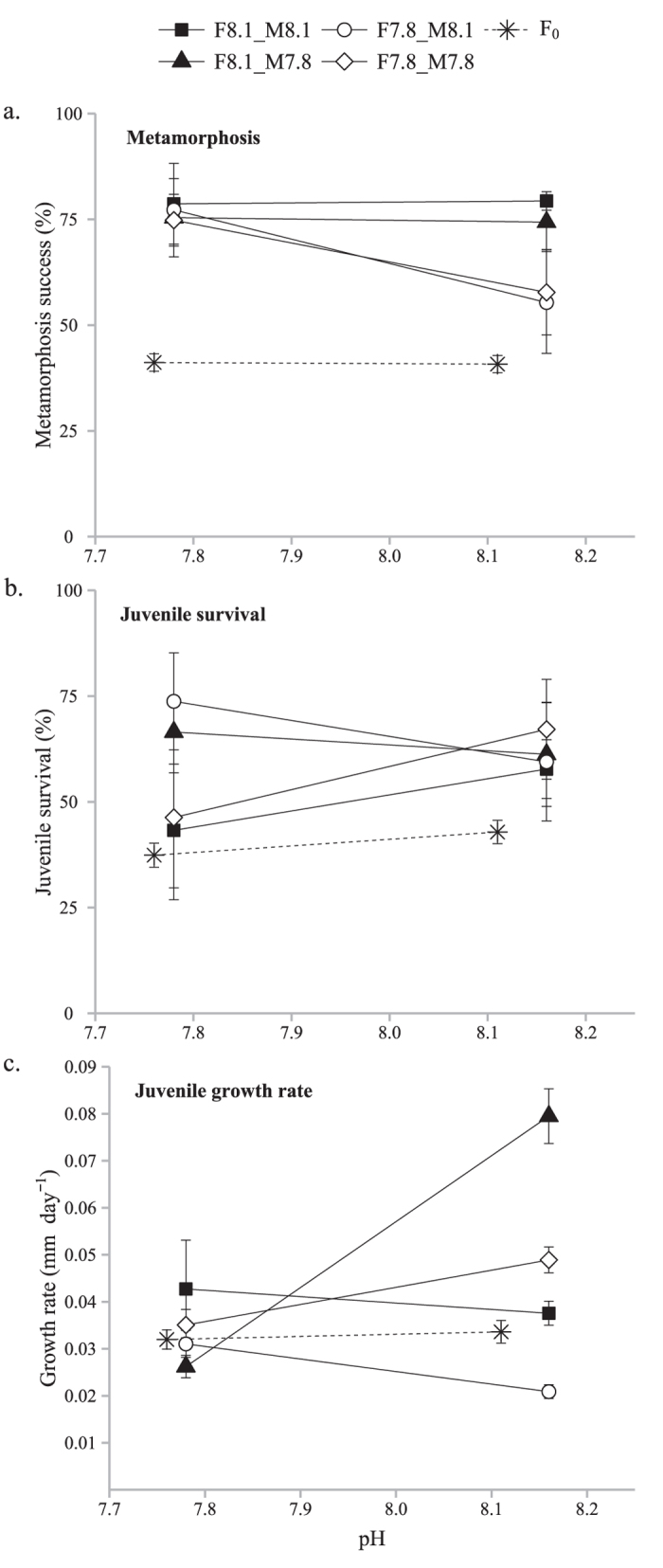
**Metamorphosis success (a, in %), juvenile survival (b, in %) and growth rate (c, in mm day^−1^) of the second generation (F_1_) of Hydroides elegans raised in two pH levels.** F_1_ groups are defined by the pH environment (8.1 vs 7.8) in which each of their parents were raised (F = maternal environment, M = paternal environment): group 1 = F8.1_M8.1; group 2 = F8.1_M7.8; group 3 = F7.8_M8.1; and group 4 = F7.8_M7.8. Mean values are expressed with their standard error of means (mean ± SEM) and the significance level applied was 5%.

For the 2^nd^ generation exposed to 2 pH environments (F_1_), 9 males and 9 females were randomly selected from all the F_0_ individuals who spawned and crossed to create four groups of egg and sperm combinations: group 1 - pH 8.1 female × pH 8.1 male (F8.1_M8.1), group 2 - pH 8.1 female × pH 7.8 male (F8.1_M7.8), group 3 - pH 7.8 female × pH 8.1 male (F7.8_M8.1), and group 4 - pH 7.8 female × pH 7.8 male (F7.8_M7.8). The four groups of fertilized embryos (F_1_ generation) were then each divided among high pH (8.16) and low pH (7.78) environments (Table 1) with (4 offspring groups × 2 pH treatments × 6 replicates = 48 total tanks) and raised from fertilization to 7 days post-metamorphosis to observe performance. Culture tanks, larval densities and food concentrations were all the same as those used for the F_0_ generation. The experimental design (spawning, pH environments, eggs and sperm crosses) is summarized in Fig. 1.

Three performance endpoints were measured for the F_0_ and F_1_ generations: (1) metamorphosis, (2) juvenile survival and (3) juvenile growth. (1) Percent metamorphosis success was calculated as the percentage of larvae successfully metamorphosing within 3 days of attaining competency. Upon larval competency, glass microscope slides with natural biofilms developed for 1 week were introduced into culture tanks and cultures were maintained for 3 days as normal. After the 3 day metamorphosis period all animals settled on the tank and slide were counted and all larvae still swimming were counted and removed. (2) Percent juvenile survival was calculated as the percentage of attached and calcifying juveniles after the 3 day metamorphosis assay that remained alive for a further 7 days under the same conditions as those during the larval development and metamorphosis periods. (3) Juvenile growth rate (mm day^−1^) was measured for the first 7 days post metamorphosis by image analysis comparing pictures taken just after the metamorphosis period to those taken 7 days later (ImageJ). Replicates with less than 10 juveniles were not considered for calculation of survival and metamorphosis, and growth rates were only calculated for replicates with more than 3 juveniles. Data were analyzed n-ways GLM models in the SAS/STAT software (SAS Institute 1990), and logarithmic transformations were used where appropriate.

To measure the experimental carbonate chemistry pH_(NBS scale)_, temperature and salinity were measured daily and total alkalinity (A_T_, Apollo SciTech AS-ALK2) was measured in triplicate for all seawater before use. The two *p*CO_2_ environments were created using continuous flow of ambient air with or without gaseous CO_2_ enrichment into environmental chambers into which replicate tanks were placed and allowed to equilibrate. Carbonate chemistry was calculated using the program CO_2_SYS (Pierrot, Lewis & Wallace 2006, doi : 10.3334/CDIAC/otg.CO2SYS_XLS_CDIAC105a) and resulted in high pH (8.11) and low pH (7.76) for the F_0_ generation and high pH (8.16) and low pH (7.78) for the F_1_ generation environments with temperature = 24 °C, salinity = 32 ppt and A_T_ = 2.28 mM and temperature = 24 °C, salinity = 33 and A_T_ = 2.24 mM respectively (full carbonate chemistry calculation reported in Table 1).

## Additional Information

**How to cite this article**: Lane, A. *et al.* Trans-generational responses to low pH depend on parental gender in a calcifying tubeworm. *Sci. Rep.* **5**, 10847; doi: 10.1038/srep10847 (2015).

## Figures and Tables

**Table 1:** Carbonate chemistry measurements and calculations for the F_0_ and the F_1_ generations’ environment.

	**Measured (±S.D.)**				**Calculated (CO_2_ sys)**				
	**pH**	**Temp (°)**	**Salinity**	**TA (mM)**	***p*CO_2_**	**CO_3_^−2^**	**CO_2_**	**Ω_Ca_**	**Ω_Ar_**
F_0_ high pH	8.11 (±0.04)	23.8 (±0.4)	32.7 (±0.6)	2.2865 (±0.113)	493.2	172.4	14.6	4.2	2.8
F_0_ low pH	7.76 (±0.04)	24.0 (±0.4)	32.7 (±0.6)	2.2865 (±0.113)	1231.4	86.2	36.2	2.1	1.4
F_1_ high pH	8.16 (±0.08)	23.7 (±0.5)	33.7 (±0.7)	2.224 (±0.061)	412.4	187.1	12.1	4.5	3.0
F_1_ low pH	7.78 (±0.04)	23.8 (±0.5)	33.6 (±0.7)	2.224 (±0.061)	1127.4	88.8	33.1	2.2	1.4

Calculated parameters were obtained using the CO_2_SYS program using the default dissociation constants and the NBS pH scale^5^.
